# Earlier Migration Timing, Decreasing Phenotypic Variation, and Biocomplexity in Multiple Salmonid Species

**DOI:** 10.1371/journal.pone.0053807

**Published:** 2013-01-10

**Authors:** Ryan P. Kovach, John E. Joyce, Jesse D. Echave, Mark S. Lindberg, David A. Tallmon

**Affiliations:** 1 University of Alaska Fairbanks, Biology and Wildlife Department, Institute of Arctic Biology, Fairbanks, Alaska, United States of America; 2 Auke Bay Laboratories, Alaska Fisheries Science Center, National Oceanic and Atmospheric Administration, Juneau, Alaska, United States of America; 3 University of Alaska Southeast, Biology and Marine Biology Program, Juneau, Alaska, United States of America; University of California, Berkeley, United States of America

## Abstract

Climate-induced phenological shifts can influence population, evolutionary, and ecological dynamics, but our understanding of these phenomena is hampered by a lack of long-term demographic data. We use a multi-decade census of 5 salmonid species representing 14 life histories in a warming Alaskan stream to address the following key questions about climate change and phenology: How consistent are temporal patterns and drivers of phenology for similar species and alternative life histories? Are shifts in phenology associated with changes in phenotypic variation? How do phenological changes influence the availability of resource subsidies? For most salmonid species, life stages, and life histories, freshwater temperature influences migration timing – migration events are occurring earlier in time (mean = 1.7 days earlier per decade over the 3–5 decades), and the number of days over which migration events occur is decreasing (mean = 1.5 days per decade). Temporal trends in migration timing were not correlated with changes in intra-annual phenotypic variation, suggesting that these components of the phenotypic distribution have responded to environmental change independently. Despite commonalities across species and life histories, there was important biocomplexity in the form of disparate shifts in migration timing and variation in the environmental factors influencing migration timing for alternative life history strategies in the same population. Overall, adult populations have been stable during these phenotypic and environmental changes (λ ≈1.0), but the temporal availability of salmon as a resource in freshwater has decreased by nearly 30 days since 1971 due to changes in the median date of migration timing and decreases in intra-annual variation in migration timing. These novel observations advance our understanding of phenological change in response to climate warming, and indicate that climate change has influenced the ecology of salmon populations, which will have important consequences for the numerous species that depend on this resource.

## Introduction

Along the coasts of the northern Pacific Ocean, salmonids (*Oncorhynchus* and *Salvelinus* spp.) are a vital link between marine and terrestrial ecosystems, and provide a massive source of nutrients to coastal food webs [1], [2]. Salmonids are also harvested for commercial, recreational, and subsistence purposes and are a sustainable fishery in Alaska [3] that has supported human coastal communities and cultures for millenia. An important aspect of salmonid biology that may need to respond to climate change is migration timing because this trait is closely adapted to local environmental conditions, particularly temperature [4], and influences individual fitness by affecting survival and reproductive success [5]–[8]. Due to their important ecological role [1], [2] and predictable migratory timing [9], many species are thought to have adapted their phenologies to correspond with the presence of adult salmon in freshwater [10], [11]. Thus, changes in this trait may have substantial ecological ramifications [1], [2], [12], and understanding the response of salmonids to climate change is imperative for conserving functional coastal human and ecological communities [2], [6]. Here, we use a multi-decade census to identify key patterns and processes in migration timing across multiple salmonid species, life histories, and life stages. In so doing, we address important research gaps concerning the phenological responses of organisms to climate change.

In addition to other factors such as harvest [13] or hatchery supplementation [14], reports of changes in migration timing for salmon species, including pink (*O. gorbuscha*), sockeye (*O. nerka*), and Atlantic salmon (*Salmo salar*) [8], [13], [15]–[19], may be due to environmental change, including climate warming [8], [17], [19]. Previous studies have been limited to single species, providing little opportunity for comparison between species and alternative life history forms in the same species, which could help resolve confounding explanations for factors affecting timing or highlight important inter- and intra-population variation.

Recent evidence suggests phenological trends among similar species occupying the same habitats can differ substantially [20]. The degree to which this holds true within and across populations as a result of different life history strategies is unknown. Intra-population heterogeneity in response to climate change could potentially be an important aspect of biocomplexity [3], especially for species such as Pacific salmon where individuals pursuing alternative life histories use different habitats and respond differently to environmental variation [9]. Similarly, quantifying the relationship between environmental variation and phenological variation for multiple species and life histories occupying the same habitats is rarely performed but may provide insight into biocomplexity within species and across populations (e.g. [3]). On a larger scale, understanding how these changes influence ecosystem processes and ecosystem services remains a critical research gap [21]. Phenotypic changes can have substantial ecological impact [22], particularly for species such as salmon that act as key components and drivers of ecosystem dynamics [1], [23].

Temporal trends in the mean/median of phenological events have been well documented for many species (e.g. [24]–[26]). But it is unknown whether there are trends in phenotypic variation (*V*_P_) within populations and if trends in *V*_P_ are independent of, or correlated with, changes in the mean phenotype [27]. Changes in *V*_P_ may be caused by shifts in allele frequencies [28], intra-generation selection [23], or environmentally dependent trait expression (i.e. plasticity, [29]). Ultimately, *V*_P_ is the basis for evolutionary change and is necessary for long-term persistence under changing environmental conditions [30], making it a critical but neglected aspect of ecological and conservation research. Because salmonid migration timing has a high heritability [31] and directional selection decreases *V*_P_ [28], we predicted that directional changes in migration timing would be correlated with decreases in *V*_P_. Alternatively, increased phenotypic variation can be evidence of exposure to novel environmental conditions and the expression of increasingly diverse phenotypes as a result of plasticity [29].

Using a 30–47 year census at a permanent weir, we describe patterns in migration timing for five species of salmonids: pink salmon, coho salmon (*O. kisutch*), sockeye salmon, Dolly Varden char (*Salvelinus malma*), and coastal cutthroat trout (*O. clarkii clarkii*). Considerable life history variation (e.g. age at maturity, age at migration to saltwater, semelparity vs. iteroparity) exists within and among these salmonid species (See Text S1, [9]), and data for different life stages (adult vs. juvenile) provide a unique opportunity to understand whether responses are consistent across life history strategies within a species. Using these data we address a number of questions: (1) has inter-annual migration timing changed in those salmonid species and life histories occupying Auke Creek, Alaska, USA; (2) what environmental or life history factors appear to play a role in determining migration timing; (3) are there temporal trends in *V*_P_ in migration timing, and, if so, (4) are these trends correlated with changes in the average phenotype; (5) how variable are phenological responses and the environmental factors influencing phenology across different species, life-stages, and life-histories; and (6) have changes in the central tendency and variance in migration timing altered the availability of salmon as an ecosystem service?

## Methods and Materials

### Study Site

**Figure 1:**
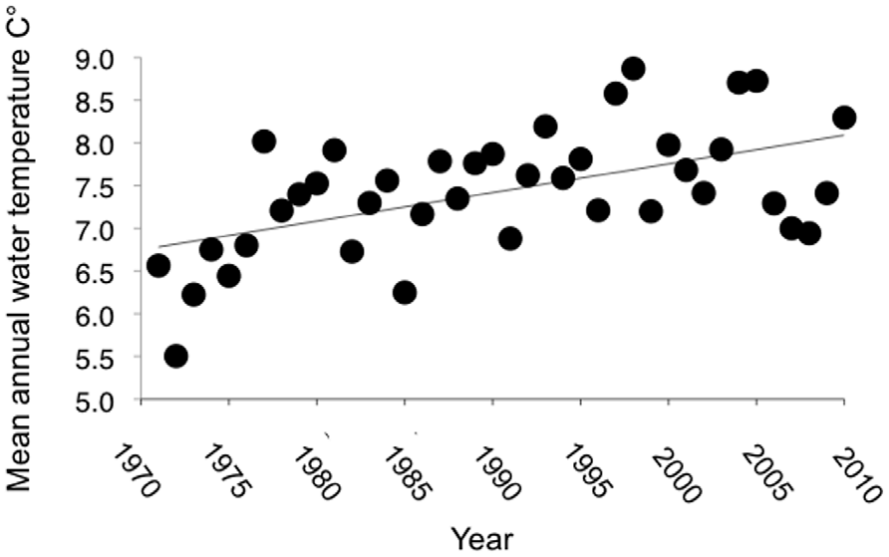
Mean annual water temperature in Auke Creek Alaska.

Auke Creek is a small, lake-outlet stream near Juneau, Alaska (Fig. S1) that has undergone rapid warming since 1971 (Fig. 1). In 1980, a permanent weir was constructed on Auke Creek just above the Auke Bay high tide mark. The weir is a National Oceanic and Atmospheric Administration (NOAA) facility, and NOAA employees conduct and oversee operations. All immigrating and emigrating salmonids are captured and counted at the weir. Prior to 1980, pink salmon juveniles were captured with fyke nets, while adult salmon (coho, sockeye and pink) were captured with a semi-permanent gated weir. While all individual fish have been counted since 1980, in years prior to 1980 very high stream flows may have reduced the efficiency of the traps in some instances (but only for coho salmon). However, this source of error was likely very small (only a few fish were undetected), and the trends in coho migration timing are robust to deleting the pre-1980 data. Therefore, a complete census is available for all individuals beginning in 1980, with some species and life histories having datasets spanning up to 47 years in length (See Text S1 for additional details on study populations). Figure S2 depicts historical and recent annual temperature profiles for Auke Creek and dates of migration timing for each species.

Because these data were collected over a 50-year time frame, much of this work predates animal care policies. The University of Alaska’s Institutional Animal Care and Use Committee (IACUC) has approved the collection of recent data under a variety of different protocols. Additionally, the weir is required to operate under a Fish Resource Permit from the Alaska Department of Fish and Game, a permitting process by which the State of Alaska ensures that research does not adversely effect fish populations. The annual operational plans are essentially unchanged, and therefore the historical data were collected in a manner that complies with contemporary animal care policies. All individual fish were captured, counted, and released unharmed either upstream or downstream of the permanent weir depending on the direction of their migration.

### Data Analyses

Linear regression was used to estimate trends in the median date of migration timing and to measure how *V*_P_ has changed over time. For the latter analysis, the response variable was the number of days over which the central 95% of migrating fish returned to or migrated out of Auke Creek. We estimated trends for each species, each life stage (juvenile vs. adult), and each life history type separately. Life history variation refers to fish with alternate life history schedules. Specifically, some reproductively mature male coho and sockeye salmon spawn at least one year earlier than all other adult salmon of either sex and are much smaller in body size (See Text S1). Although these are reproductively mature fish, we refer to them separately as “jacks” to differentiate them from non-jack adults, which we refer to as simply “adults”. Another source of life history variation occurs within juvenile sockeye and coho salmon, where fish can migrate to the ocean after spending 1 or 2 years in freshwater. We refer to these fish as age 1 or age 2 smolts. Finally, there are both odd- and even-year pink salmon populations in Auke Creek (Text S1), and we estimated trends in migratory timing for these populations separately.

Table S1 provides the sample size (number of years) for each species and life history as well as the estimates from the linear regression of migration timing and *V*_P_ vs. year. To quantify the relationship between temporal trends in the median date of migration timing and *V*_P_, we calculated the correlation between responses across all species and life histories using Spearman’s rank correlation. In order to directly test the hypothesis that larger temporal trends in migration timing are positively correlated with decreasing *V*_P_, we standardized the estimates of the temporal trends by taking the absolute value of each response, calculated a mean of the absolute values, and then subtracted this mean value from each absolute value (
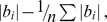
 where the *b*_i_ are the estimates of change in migration timing and *n* is the total number of estimates).

We used several analyses to describe temporal changes in the distribution of adult salmon in freshwater. We estimated the change in the peak period that adult salmon are available in Auke Creek by performing a linear regression of the difference between the median dates of adult coho and sockeye salmon migration vs. year (i.e. the earliest and latest migrating species respectively). An identical analysis was performed on the number of days between the first and last 100 salmon to enter freshwater as a response variable. Finally, linear regression was used to estimate the temporal trend in the cumulative number of days that adult salmon migrate into Auke Creek. For the response variable, we summed across pink, coho and sockeye salmon the number of days over which the central 95% of migrating fish returned to Auke Creek in each year. We did not include jacks in these analyses. We performed identical analyses for migration timing from freshwater to the ocean for juvenile salmon, but we used the first and last 1000 fish to leave freshwater as a response variable because juvenile abundances are much larger. For these analyses, we combined juvenile sockeye and coho because these fish have largely overlapping migratory distributions.

We estimated population growth rate for adult salmon species (coho, sockeye, and pink) because the interaction between abundance and migration timing constrains the harvest allotted to fisheries and the impact of salmon on ecosystems through bioturbation and marine-derived nutrient subsidies [1], [2]. We used the exponential growth state-space method [32] to estimate population growth rate from 1971–2010 for adult pink, sockeye and coho (1971–2009; data from 2010 weren’t available when analyses were conducted) salmon, and we report discrete-time estimates (i.e. lambda). This method estimates population growth rate based on the observed numerical abundances of adult salmon across time. We did not calculate population growth rates for cutthroat trout or dolly varden from Auke Lake because these are mixed-stock populations composed of individuals that spawn in different locations (See Text S1), which could result in misleading estimates.

**Figure 2:**
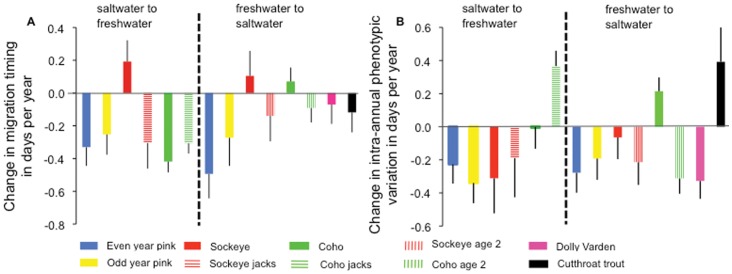
Change in migration timing for salmonids in Auke Creek, AK. (a) Temporal change in median date of migration timing. The color key for the different species is located below the figures. For both panels, the error bars represent standard errors, and the dashed line separates estimates of temporal trends in migration timing from saltwater to freshwater (left side), from temporal trends in migration timing from freshwater to saltwater (right side). (b) Change in intra-annual variation in migration timing (*V*_P_ ) estimated from linear regression of the number of days over which each life history type migrates into or out of Auke Creek vs. year.

We used an information theoretic approach based on Akaike’s Information Criterion adjusted for sample size (AIC_C_) to identify models that best described inter-annual variation in migration timing for the various species and life histories in Auke Creek [33]. We used simple linear regressions with *a priori* defined covariates that we hypothesized influenced migration timing (Fig. S3, See Text S1). Median date of migration timing for each species/life history was used as the response variable. For these analyses, we combined data from even- and odd-year pink salmon to increase sample size, given that these populations demonstrated similar temporal trends in migration timing (Fig. 2), and should respond somewhat similarly to inter-annual environmental variation.

A variety of environmental variables, some of which have been monitored in Auke Creek (stream temperature and precipitation/streamflow), were used in these analyses (Text S1). We also included variables representing biological (density) and oceanic conditions (Pacific Decadal Oscillation and sea surface temperature, Text S1). We standardized predictor variables by subtracting the mean from each value and dividing by the standard deviation. The importance of each predictor variable was compared by summing the AIC_C_ weights from each model that included a given covariate and had a ΔAIC_C_<10 [33], [34]. Identical candidate model sets (Table S2) were used for all reproductively mature fish, including jacks, migrating into Auke Creek to facilitate comparison between species and life histories. Similarly, we used identical candidate model sets (Table S3) for all fish migrating from Auke Creek to the ocean; but we did not include density in models for pink salmon because juvenile density interactions in freshwater are negligible for this species. We primarily considered additive models, but included interactive effects where we hypothesized they may be important. Specifically, we hypothesized that the influence of water temperatures on migration timing may have changed over time due to temporal shifts in migration timing and/or increasing water temperatures. This could arise from novel or increased selective pressures on migration timing, or different patterns of phenotypic plasticity in novel environments [29]. For example, juvenile salmonids may have historically migrated to the ocean earlier when freshwater temperatures were warmer, but early migration timing as a result of increasingly warm freshwater temperatures may be disadvantageous, resulting in a selective advantage for fish not responding to increasing stream temperatures (e.g. [6]).

In addition to environmental variables, we included time (year) as a continuous covariate in some models (See Text S1 for additional details on study populations). We included year as a surrogate for unmeasured environmental change and/or as a variable representing directional evolutionary change in migration timing, which has been observed in other salmon populations [35]. We tested covariates for pair-wise correlations (i.e. multicollinearity; Table S4), and found that our candidate variables were minimally correlated [33], [36]. All data analyses were performed in R (The R Development Core Team 2010).

## Results

We observed earlier migration timing from saltwater to freshwater (Fig. 2A) in odd-year pink salmon adults (*b*_1_ (i.e. slope of the regression) = −0.253, *SE* = 0.12), even-year pink salmon adults (*b*_1_ = −0.331, *SE* = 0.11), coho salmon adults (*b*_1_ = −0.418, *SE* = 0.07), coho salmon jacks (*b*_1_ = −0.307, *SE* = 0.06), and sockeye salmon jacks (*b*_1_ = −0.305, *SE* = 0.16) but not sockeye salmon adults (*b*_1_ = 0.192, *SE* = 0.13). Migration from freshwater to the ocean showed a similar shift toward earlier timing (Fig. 2A), as estimates for trends in migration timing for Dolly Varden (*b*_1_ = −0.070, *SE* = 0.12), cutthroat trout (*b*_1_ = −0.119, *SE* = 0.12), odd-year pink salmon (*b*_1_ = −0.494, *SE* = 0.15), even-year pink salmon (*b*_1_ = −0.273, *SE* = 0.17), age 2 sockeye salmon (*b*_1_ = −0.14, *SE* = 0.16), and age 2 coho salmon (*b*_1_ = −0.091, *SE* = 0.09) were all negative. However, trends in migration timing for age 1 coho (*b*_1_ = 0.070, *SE* = 0.09) and age 1 sockeye (*b*_1_ = 0.105, *SE* = 0.15) were positive. *V*_P_ in migration timing has decreased for many species and life history types (11 of 14, Fig. 2B), but the magnitude of temporal trends in the median date of migration timing was not correlated with decreases in *V*_P_ (Spearman’s Rank *r = *0.055, *P* = 0.852).

**Figure 3:**
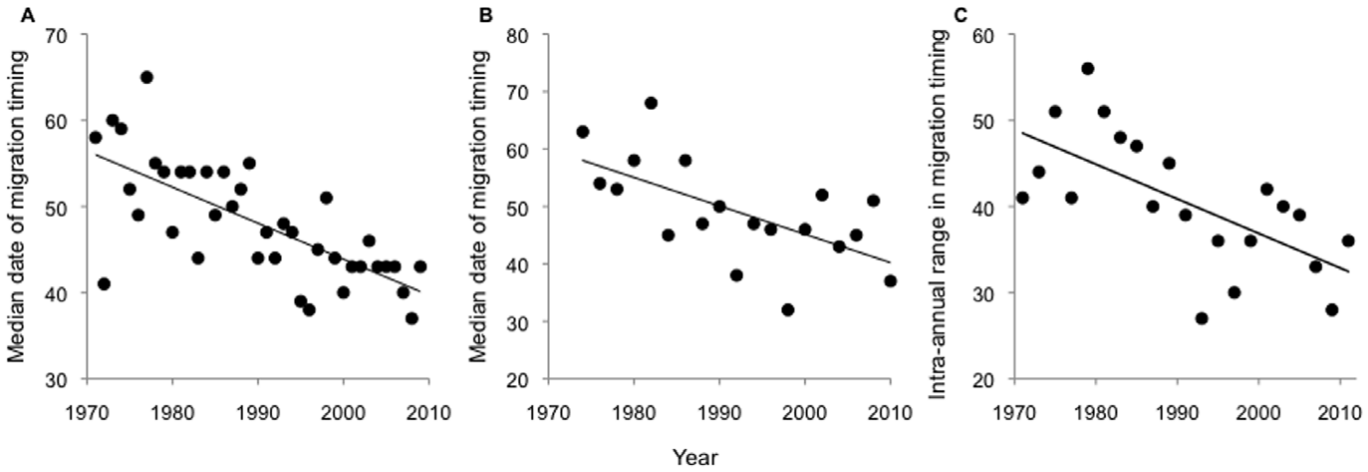
Examples of temporal changes in the median date of migration timing and *V*_P_. These plots show the greatest trends toward earlier migration timing from saltwater to freshwater (adult coho, A), freshwater to saltwater (juvenile odd-year pink salmon, B) and decreasing intra-annual range in migration timing (adult odd-year pink salmon, C). The lines are the fitted regressions. Please note that odd-year (referring to year of adult spawning) juvenile pink salmon migrate in even years.

The greatest change in migration timing from saltwater to freshwater was for adult coho salmon (Fig. 3A), which are now migrating into Auke Creek approximately 17 days earlier than they did 40 years ago. Freshwater temperatures during migration were positively related to migration timing for all species, except for sockeye jacks (*b*_1_ = −4.16, *SE* = 2.54). Similarly, there was a positive relationship between migration timing and the date of peak stream flow during the migration for all species and life histories except for sockeye jacks (*b*_1_ = −1.198, *SE* = 1.92). Therefore, most individuals appear to avoid migrating during high temperatures and low flows (i.e. migrate during high flows and cooler temperatures). Overall, there was earlier migration from saltwater to freshwater in all species and life history types except for adult sockeye salmon (Fig. S3, Table 1 and S2).

**Table 1:** Parameter estimates from the best-supported model for migration timing from saltwater to freshwater for each species and life history type.

	Parameter					
Species	Y	T	P	S	F	R^2^
Pink salmon	−0.34 (0.07)	3.13 (0.86)				0.45
Coho adults	−0.37 (0.07)	1.61 (0.74)			1.75 (0.82)	0.64
Coho jacks	−0.29 (0.06)			1.48 (0.71)		0.45
Sockeye adults		3.35 (1.64)	4.10 (1.64)	−2.67 (1.74)	3.35 (1.68)	0.30
Sockeye jacks	−0.31 (0.16)					0.09

Standard errors for each estimate are in parentheses. Labels for the covariates are Year = year, T = temperature during peak migration, P = Pacific Decadal Oscillation, S = sea surface temperature, F = stream flow. All covariates are standardized except for Year.

Although there were strong relationships between stream temperatures and migration timing for mature salmon, other variables were also important. Interactive terms between year and stream flow and year and temperature were important for both sockeye jacks and coho jacks respectively. However, a model for coho jacks that included year and sea-surface temperature was equally supported (ΔAIC_C_ = 0.001), and is most parsimonious (2 vs. 3 predictor variables). In Table 1, we present estimates from the more parsimonious models. For sockeye jacks, a model that only included year was well supported by the data (ΔAIC_C_ = 0.7) and had two fewer predictor variables (3 vs. 1) than the interactive model. Oceanic conditions were included in those models with the lowest AIC_C_ for pink salmon, sockeye adults, and coho jacks (Table 1). However, the model that did not include PDO for pink salmon was equally supported by the data (ΔAIC_C_ = 0.04), and the variables for year and stream temperatures during migration had much more support than oceanic conditions when compared across models (Fig. S3). Sea-surface temperatures had opposite relationships with migration timing for sockeye adults and coho jacks; sockeye migration timing was negatively related to sea surface temperature but the opposite was true for coho jacks (Table 1). Finally, there was intra-specific variation in phenological trends for sockeye salmon, where alternative life histories (adults and jacks) show contrasting temporal trends in migration timing. Adult sockeye are migrating later in the year, while sockeye jacks are migrating earlier (Fig. 2).

Migration timing from freshwater to the ocean was strongly related to stream temperature and stream temperatures are increasing (Fig 1A, Fig. S3, Table 2, Table S3). In the most extreme case, odd-year pink salmon juveniles are now leaving Auke Creek for the ocean approximately 19 days earlier than in 1974 (Fig. 3B). Stream temperature during peak outmigration to the ocean was the best overall predictor, and had a negative relationship with timing of outmigration for all juvenile Pacific salmon, cutthroat trout, and char (Fig. 2, Table 2 and S3). Dolly Varden and cutthroat trout were the most sensitive to variation in water temperature in terms of their migration timing. Based on the most supported model predicting migration timing, dolly varden and cutthroat trout migrate into freshwater 4.62 (*SE* = 0.67) and 4.9 (*SE = *0.67) days earlier (respectively) for each 0.506°C (one standard deviation in the variability of stream temperatures during trout and char migrations) increase in temperature. Cumulative stream temperatures during the growth period from the previous year (or incubation period for pink salmon) did not appear to be strongly related to migration timing, but there was a detectable effect for age 2 coho and age 1 sockeye salmon (warmer temperatures were associated with earlier dates of migration timing). Despite the commonalities, age 1 and 2 juveniles of both sockeye and coho salmon demonstrate contrasting trends in migration timing; in both species age 2 juveniles make up an increasing proportion of the outmigrants (Fig. S4) and are migrating earlier, whereas age 1 juveniles are decreasing in relative abundance and migrating later.

**Table 2:** Parameter estimates from the best-supported model for migration timing from freshwater to saltwater for each species and life history type.

	Parameter					
Species	Y	T	Tlag	D	Y*Tlag	R^2^
Pink salmon	−0.29 (0.07)	−6.43 (0.74)				0.76
Coho age 1		−2.54 (0.57)		−1.08 (0.56)		0.48
Coho age 2		−3.22 (0.46)	−1.05 (0.52)			0.75
Sockeye age 1	0.09 (0.14)		−13.53 (4.55)		0.59 (0.18)	0.33
Sockeye age 2		−3.90 (1.20)				0.27
Dolly Varden		−4.62 (0.67)				0.62
Cutthroat trout		−4.90 (0.67)				0.65

Standard errors for each estimate are in parentheses. Labels for the covariates are Year = year, T = temperature during peak migration, Tlag = temperature during the previous year (a lagged effect of temperature) or incubation period (pink salmon), D = conspecific density. Models are additive unless specified otherwise (e.g. Y * Tlag). All covariates are standardized except for Year.

There was also a strong relationship between juvenile pink salmon migration timing and freshwater temperature, where pink salmon migrate into saltwater nearly one week earlier for each 1.33°C (one standard deviation) increase in water temperature. Generally, year was a poor predictor of migration timing from freshwater to the ocean, but there was a negative linear trend in pink salmon migration timing. As noted elsewhere [19], this trend is partially due to changes in adult migration timing, because earlier adult spawning can result in an earlier juvenile outmigration the following spring. Nonetheless, when adult migration timing from the previous year (i.e. the parental generation; *b*_1_ = 0.323, *SE* = 0.11) is included in the most supported model predicting migration timing of pink salmon from freshwater to saltwater (Temperature+Year+Adult migration timing), year remains an important variable in the model based on effect size (*b*_1_ = −0.210, *SE* = 0.07). This *post-hoc* model [37] also had more support (AIC_C_ = 218.931) than a model that included adult migration timing in the previous year, but not year itself (Temperature+Adult migration timing; AIC_C_ = 226.869). In other words, the trend toward earlier migration timing for juvenile pink salmon does not appear to be due to changes in adult migration timing.

The estimated trends in *V*_P_ indicate that 11 of 14 salmonid life histories are migrating over a shorter range of dates. In the most extreme case of decreasing *V*_P_, odd-year adult pink salmon are now migrating over a period of time that is on average 13 days shorter (from 46 to 33 days) than it was forty years ago (Fig. 3C, *b*_1_ = −0.340, *SE* = 0.12). The average response across all species and life histories was a 10.2% decrease in *V*_P_ (*SE = *6.1%), and the greatest single change was a 41% decrease in *V*_P_ for age 2 sockeye salmon. Alternative life histories in coho salmon (in both adults and juveniles) had contrasting trends in *V*_P_. Coho jacks (*b*_1_ = 0.366, *SE* = 0.09) and age 1 smolts (*b*_1_ = 0.214, *SE* = 0.08) are both migrating over a longer period of time, while age 2 smolts (*b*_1_ = −0.192, *SE* = 0.13) are migrating over a shorter period of time, and coho adults (*b*_1_ = −0.002, *SE* = 0.13) have demonstrated almost no change in the duration of their migration. Excluding even-year juvenile pink salmon, there was little evidence for correlations between numerical abundance and the number of days that fish migrate into or out of freshwater (Pearson’s *r* = −0.401–0.449). The number of days over which even-year juvenile pink salmon migrated into saltwater was positively correlated with abundance (Pearson’s *r* = 0.769). However, there was no temporal trend in the natural logarithm of juvenile pink salmon abundance (*b*_1_ = 0.045, *SE* = 0.04), suggesting that the trend in *V*_P_ for this life history is independent of the effects of abundance (i.e. the trend in *V*_P_ is not due to a change in abundance).

As a result of the changes in median dates of migration timing, the range of dates over which adult salmon return to spawn in Auke Creek and are available as a resource in freshwater has decreased from 79 to 55 days (*b*_1_ = −0.618, *SE* = 0.18). The decrease in the range of dates over which all adult salmon return to freshwater is even greater when considering the number of days between the first 100 and last 100 salmon to enter freshwater (*b*_1_ = −0.697, *SE* = 0.14). Finally, the cumulative number of days per year that adult salmon migrate past the weir into freshwater has decreased (*b*_1_ = −0.792, *SE* = 0.23) by approximately 31 days. The window of time over which juvenile salmon migrate out of Auke Creek has shown less change over time, or has even increased. Specifically, the range of dates over which fish migrate out of Auke Creek has increased from 30 to 40 days (*b*_1_ = 0.340, *SE* = 0.15) based on median dates of migration timing from 1980–2010. The number of days between the first and last 1000 juvenile salmon to exit Auke Creek has marginally increased (*b*_1_ = 0.232, *SE* = 0.23), while the cumulative number of days over which the majority of fish migrate past the weir has slightly decreased (*b*_1_ = −0.173, *SE* = 0.16).

Overall, the abundances of adult salmon have not decreased and population growth rates are stable. We observed that population growth rate (λ) was close to the replacement level of 1.0 and the associated 95% confidence intervals (CI) all encompassed 1.0 for odd-year pink salmon λ = 1.064 (0.740,1.530), even-year pink salmon λ = 1.059 (0.700,1.603), sockeye salmon λ = 0.971 (0.853,1.106), and coho salmon λ = 0.997 (0.969, 1.027).

## Discussion

Our results show that the migration timings of Auke Creek salmonids have changed across species and life history types over the last 30–47 years. In general, fish are migrating earlier (11 of 14 species, life stages, and life histories) and over a narrower range of dates (11 of 14 species, life stages, and life histories). Although the temporal availability of adult salmon has been reduced, their abundances remain stable. Earlier migration timing of salmon from saltwater to freshwater is opposite to what we would predict given that adult salmon in Auke Creek migrate in the fall and prefer cooler water temperatures (Table 1). Earlier migration timing has also been observed in Columbia River, Fraser River, and Bristol Bay sockeye salmon [13], [15], [16]. Environmentally induced changes in salmon migration timing have now been observed across the northern Pacific Ocean, raising the possibility that these general patterns may be due to large-scale environmental change such as climate warming. The generality of shifts toward earlier migration timing across species, life stages, and life histories within this study location is indicative of biological response to a common phenomenon. While changes in migration timing have been implicated in reduced fitness in some salmonid populations [15], [38], populations of adult salmon in Auke Creek have been stable.

At our study location, the only species not migrating earlier were adult sockeye. Unlike pink and coho, sockeye do not reproduce immediately after entering Auke Creek, but instead mature in Auke Lake for up to a month before spawning in August [39]. As a result, they appear to have more plasticity in their migration timing (*P*<0.001, pair-wise *F* tests for equality of inter-annual variation in migration timing), and appear to be more strongly influenced by local environmental variation, particularly stream flows, which can be prohibitively low during peak periods of sockeye migration. Whether Auke Creek sockeye are actually reproducing earlier in time is unknown.

Migration timing in salmon populations can also be influenced by other factors including salinity [40], [41], harvest [13], or hatchery activities [14]. In Auke Creek, hatcheries have not had persistent directional effects on these populations because augmentation occurred over brief periods of time for pink salmon (1973–1980, 1989, 1996) and sockeye salmon (1988–1992). In years with hatchery returns, the percentage of hatchery fish relative to wild fish varied widely for both sockeye (1%−61%; mean = 33%) and pink salmon (1–71%; mean = 34% for odd year, 55% for even year), We excluded hatchery fish from our analyses, but they were allowed upstream of the weir and could have spawned with wild fish. The effects of commercial fisheries are much more complicated in Southeast Alaska than in other well studied areas (e.g. Bristol Bay [13]) because of the immense variability in the geography and biology of the salmon populations in this region [42], and the fact that commercial harvest does not occur at or anywhere near the mouth of Auke Creek. Harvest rates are not known for pink and sockeye populations (sockeye harvest has been closed in Auke Bay since 1980), but commercial fisheries appear to harvest approximately 40% of Auke Creek coho [43]. For all species, the fisheries occur on mixed stocks migrating through distant purse seine, drift gillnet, and trolling fisheries, making it unlikely that harvest-induced directional selection on migration timing is persistent for these adult salmon populations. Simply, harvest occurs but it is more likely to be stochastic than deterministic as has been observed elsewhere (See also Text S1). For coho, this is strongly supported by the fact that we observed similar temporal trends in migration timing for jacks and adult salmon, but neither commercial nor recreational fisheries target jacks in this populations.

The fact that juvenile salmonids develop more quickly and migrate to saltwater earlier with increasing stream temperatures is well documented (e.g. [8], [44], [45] and reviewed in [46] for sockeye), and is assumed (at least historically) to be an adaptive behavior allowing juvenile salmon to exploit peak resource availability in estuarine environments (e.g. [9], [19]). Based on our results, this relationship between freshwater temperature and migration timing also holds for mature and juvenile trout and char, both of which are iteroparous. Both species appear to migrate out of Auke Creek approximately 9–10 days earlier for each 1.0°C increase in water temperature. Reproductively mature cutthroat trout that leave Auke Creek immediately move to other freshwater streams to spawn, suggesting that stream temperature may be acting as a migratory cue or is directly influencing the timing of this life history event [47]. Also, cutthroat trout and Dolly Varden can feed heavily on juvenile salmon that are migrating into saltwater, and it is believed that their migrations may be timed to coincide with the availability of juvenile salmon resources (e.g. 48]). This raises the possibility that “phenological cascades” may be occurring, or will occur, among salmon, trout, and char migrating from freshwater to saltwater. Overall, projections of rapidly warming temperatures [49] imply that substantial changes in migration timing from freshwater to saltwater are likely. Future changes in migration timing to salt water are concerning because of the potential for juvenile salmon to experience trophic mismatches in the oceanic environment, that is, arriving in the ocean before their primary food resource, zooplankton, has undergone its spring bloom [6]–[8], [19]. Alternatively, these fish may be successfully tracking resource availability if springtime blooms of zooplankton in Auke Bay are also occurring earlier in time. The latter possibility is supported by the stable population dynamics observed in adult salmon populations.

The decrease in *V*_P_ for pink salmon (all life stages), sockeye salmon (all life stages and life histories), coho salmon (age 2 smolts), and Dolly Varden, has broad implications because of the importance of phenotypic variation and biocomplexity to Pacific salmon population stability [3], [50]–[53]. Migration timing from freshwater to saltwater can strongly influence juvenile salmon survival [7], [8], [54], but optimal migration timing varies from year to year [7]. Reductions in the window of time that salmonids migrate into saltwater may decrease the probability that migration events coincide with optimal conditions. Similarly, Dolly Varden migrations to the marine environment are also occurring over a shorter period of time, and there may be some risk migrating fish will fail to coincide with peak food resources [55].

It is possible the observed phenotypic changes have influenced evolutionary and population dynamics [56]–[58], especially given the high heritability of phenology in salmonids (median *h* ^2^ = 0.51, [31]). We predicted larger decreases in *V*_P_ with greater temporal shifts in migration timing, which could be taken as evidence for directional selection and possible genetic response to this selection. Instead, we found no correlation between changes in *V*_P_ and changes in the median date of migration timing, suggesting that these two aspects of the phenotypic distribution have responded to climate warming independently. A notable exception was in odd- and even-year pink salmon, where fish from both populations are migrating earlier and over a reduced period of time. Along with these observations, genetic data indicate that there has been a genetic change for earlier migration timing in the odd-year pink salmon population [59]. The degree to which these phenotypic changes, including changes in *V*_P_, are a function of microevolution as opposed to phenotypic plasticity is a critical unknown for the other populations at Auke Creek and for the many phenological changes observed elsewhere [60]. Directional changes, coupled with high heritability, suggest that evolution by natural selection may have occurred in some of these populations, and empirical research on other salmon populations supports this possibility [35]. However, there is a strong plastic component to migration timing and development in salmonid fishes (e.g. [6], [43], [61]), and an alternative explanation is that the observed shifts are entirely due to environmentally induced trait expression [62]. In fact, the only 3 trends toward increased phenotypic variation (coho jacks, age 1 coho, cutthroat) were fairly strong, potentially indicative of novel phenotypic expression due to plasticity [29]. It may be possible to test these hypotheses with further experiments and/or analysis of temporal genetic data [63].

Despite commonalities in temporal trends in migration timing and *V*_P_, an important observation is that different life history types within a population can have disparate phenological responses to climate change (i.e. biocomplexity *sensu* [3], Fig. 2A, Fig. 2B). Opposite temporal trends among life histories within species were observed for both median date of migration timing (sockeye adults vs. jacks and age 1 vs. 2 sockeye and coho smolts) and *V*_P_ (age 1 vs. 2 coho salmon smolts). Additionally, different environmental variables influenced migration timing for different species and life histories (Fig. S3, Tables 1 and 2). The disparity between sockeye adults and jacks may be due to the positive relationship between adult sockeye migration timing and the Pacific Decadal Oscillation [64], or that sockeye jacks may be less influenced by high stream temperature and low flow due to their smaller body size [65]. This latter idea is supported by the fact that freshwater environmental variables had little influence on migration timing for sockeye jacks.

The contrasting patterns of temporal changes in phenology for age 1 and 2 sockeye and coho salmon smolts are interesting given the common effect of water temperature on these life histories. Smolts of different age classes differ in their timing of outmigration to saltwater (Text S1) and are subject to different environmental conditions (e.g. streamflow) due to rapid changes in springtime hydrological cycles [66]. In some years, age 1 smolts migrate after peak springtime flows and their migrations may be constrained by low stream flows until rain events increase stream discharge. Temporal trends toward an increasing proportion of age 2 smolts (Fig. S4) are another indicator that complex environmental and/or genetic changes may be occurring within these populations. Generally, the largest/oldest fish (age 2) tend to outmigrate earlier and have higher marine survival (e.g. [67] and see Text S1). Therefore, if selection is occurring, it may be acting on migration timing or age at outmigration, either of which could produce the observed trends [23], [68]. It does appear that selection and/or some unidentified environmental change may be leading to these shifts in timing and age structure. This is because increasing water temperatures in Alaskan lakes have been shown to increase juvenile salmon growth [69], [70], which should in turn lead to faster maturity and therefore an increasing prevalence of age 1 fish (e.g. [71]). This pattern is opposite to what we observed, suggesting some other mechanism may be influencing these populations. Regardless of process, these observations highlight the conservation value of preserving life history variation in the face of uncertainty. Our present biological knowledge is insufficient to adequately predict how climate change will impact many populations [20] and how various life histories will contribute to population growth and persistence [3], [50].

Changes in adult salmon migration at Auke Creek timing since 1971 have reduced the time period that salmon are available in freshwater as an ecosystem service and resource subsidy by nearly a month. These results have significant implications for salmon management because commercial, subsistence, and recreational fisheries are partially determined by an accurate knowledge of migration timing [13], [51]. Also, compressed migration distributions within and between salmon species could potentially increase density dependence as a result of competition for spawning areas and therefore act to decrease population abundance. Because the phenologies of other organisms are adapted to the timing of salmon spawning, these changes have the potential to influence other ecological interactions (e.g. mayfly emergence [10], plant-pollinator interactions [11], wildlife behavior [72]) and both human and non-human consumers of adult salmon will need to adjust their behavior to continue to consume Auke Creek salmon. On the other hand, the window of time that juvenile salmon migrate out to the ocean and are available as prey resources for fish (e.g. [9], [48], [73]) and wildlife (reviewed in [74]) in freshwater, intertidal, and nearshore ecosystems has remained relatively stable or even increased.

In summary, this long-term, multi-species, multi-life history data set allowed us to make several novel insights into the process of phenological change in response to climate warming for a suite of species and life history stages. By examining migration timing across multiple species and life stages, we found that salmonids in Auke Creek are generally migrating earlier during a period of environmental warming. The dramatic decrease in *V*_P_ across most life histories indicates a general trend at this study site. However, this statistic is almost never reported in other studies of phenological change (but see [27]), making it difficult to generalize to other studies [21]. Researchers should report changes in *V*_P_, given that it is a critical parameter that may indicate the long-term capacity for populations to persist during changing environmental conditions [30], [75], [76]. Importantly, a majority of the contrasting temporal trends in migration timing were intra-specific as opposed to inter-specific. Therefore, heterogeneity in phenological changes within species can be as great as variation between species occupying the same habitat. The phenological changes observed in this stream have reduced the temporal distribution and availability of an important ecosystem service and source of marine derived nutrients, a trend that will influence other aquatic and terrestrial species. At present, it seems impossible to predict how long these populations will remain stable if the observed trends in migration timing and environmental warming continue.

## Supporting Information

Figure S1 **Map of the study area in relation to Southeast Alaska and the entire state of Alaska.**
(TIFF)

Figure S2 **Average dates of salmon migration timing and average daily water temperatures (°C) for Auke Creek Alaska.** The solid line represents the average daily water temperature from 1971–1980 and the dashed line represents the average daily water temperature from 2001–2010. The average date of migration timing is labeled for each species. Alternative life histories (e.g. sockeye adults and jacks) within a species and life-stage are combined for greater clarity, and the average date of their migration timing is presented. The vertical lines from the temperature trends to the species description represent the average of the median dates of migration across the time series. Lines to the left of the dashed vertical line are for migration timing from freshwater to saltwater, and lines to the right depict migratory timing from saltwater to freshwater.
(TIFF)

Figure S3 **Relative support for variables used to predict migration timing for each species and life history.** Variables are depicted on the x-axis by Y = Year, T = Stream temperature during peak migration, P = Pacific decadal oscillation, S = Sea surface temperature, F = Peak stream flow, Tlag = Average stream temperature during the growth and development period, D = conspecific density (See SI text). The y-axis is the sum of the AIC_C_ weights from each model that included a given covariate and had a ΔAIC_C_<10, and represents the relative support for each variable. Variables with relative importance = 1 are present in all best-supported models. For each species and life history, all candidate variables are included on the figure, even those with relatively less support. See Figure 1 caption for description of figure colors (Blue = Pink salmon even- and odd-years combined). Shaded panels represent migration timing from freshwater to saltwater.
(TIFF)

Figure S4 **Proportion of age 1 smolts vs. time for sockeye (A) and coho (B).** The fitted lines are linear regressions of the proportion of smolts vs. time (A, *b*1 = −0.014, *SE* = 0.006) (B, *b*1 = −0.006, *SE* = 0. 0.002) salmon.
(TIFF)

Table S1 **Results from the linear regressions of timing of migration and intra-annual trait variation vs. year.** Regression describes which data were used in the analysis, *n* = sample size, *b* = slope from the regression, SE(*b*) = standard error of the slope, L CI = lower 95% confidence interval, U CI = upper 95% confidence interval, *r* ^2^ = coefficient of determination, *P* = *P* value of the regression analysis.
(DOCX)

Table S2 **Model selection results for migration timing from saltwater to freshwater. AIC_C_ values for the models predicting the median date of migration timing from saltwater to freshwater for reproductively mature Pacific salmon.** The model with the lowest AIC_C_ is highlighted in yellow. *Y* = year, *T* = temperature during migration, *P* = PDO, *S* = sea surface temperature, *F* = peak stream flow.
(DOCX)

Table S3 **Model selection results for migration from freshwater to saltwater.** AIC_C_ values for the models predicting the median date of migration timing from freshwater to saltwater for the various species and life histories. The model with the lowest AIC_C_ is highlighted in yellow. *Y* = year, *T* = temperature during migration, *Tlag* = temperature during the developmental period leading up to migration, *D* = conspecific density (See covariate descriptions).
(DOCX)

Table S4 **Correlations between environmental variables and time (Year) for those data used to predict migration timing for each species and life history.** All values are Pearson product moment correlations. Values above the double horizontal line are for data used to predict median date of migration timing into freshwater from the ocean, and values below are for data used to predict salmonid migration timing from freshwater into saltwater. T refers to water temperatures during peak migration timing, F refers to flows during peak migration timing, PDO refers to values of the Pacific Decadal Oscillation, SST refers to sea-surface temperature, and Tlag refers to temperatures during developmental periods in freshwater (see Text S1 for more information). The label “All” refers to data used for all species and life histories migrating into saltwater except for pink salmon.
(DOCX)

Text S1 **The supplementary text provides a description of each species life history, and a detailed description of the environmental variables used in model selection analyses.**
(DOCX)
