# Supplementary figures and images for: Earlier Migration Timing, Decreasing Phenotypic Variation, and Biocomplexity in Multiple Salmonid Species
Source: PLoS One. 2013 Jan 10;8(1):e53807. doi: 10.1371/journal.pone.0053807 (PMC3542326; doi:10.1371/journal.pone.0053807)

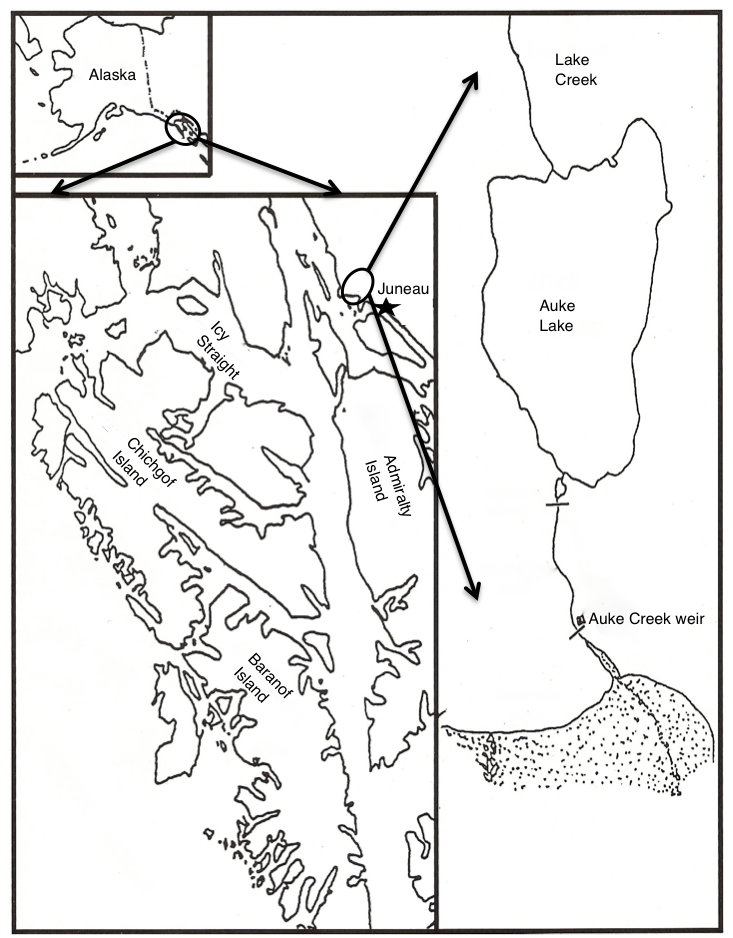

Supplement: Figure S1 — Map of the study area in relation to Southeast Alaska and the entire state of Alaska. (TIFF) [file pone.0053807.s001.tiff]

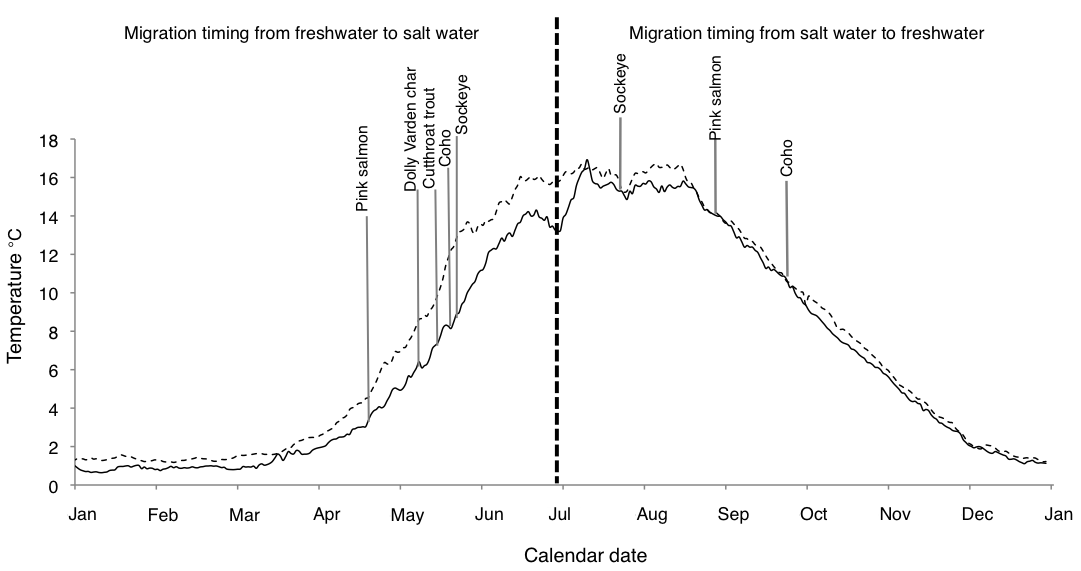

Supplement: Figure S2 — Average dates of salmon migration timing and average daily water temperatures (°C) for Auke Creek Alaska. The solid line represents the average daily water temperature from 1971–1980 and the dashed line represents the average daily water temperature from 2001–2010. The average date of migration timing is labeled for each species. Alternative life histories (e.g. sockeye adults and jacks) within a species and life-stage are combined for greater clarity, and the average date of their migration timing is presented. The vertical lines from the temperature trends to the species description represent the average of the median dates of migration across the time series. Lines to the left of the dashed vertical line are for migration timing from freshwater to saltwater, and lines to the right depict migratory timing from saltwater to freshwater. (TIFF) [file pone.0053807.s002.tiff]

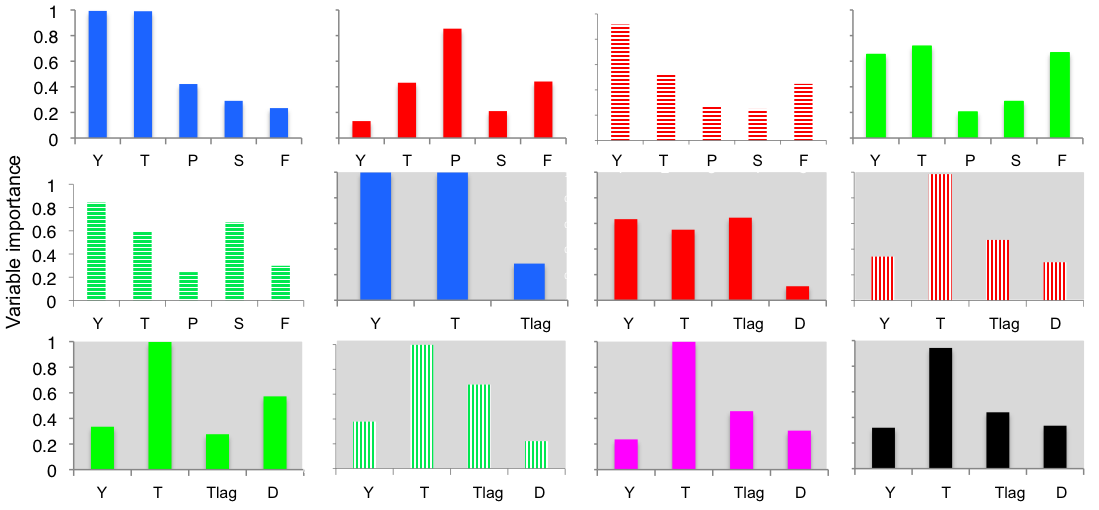

Supplement: Figure S3 — Relative support for variables used to predict migration timing for each species and life history. Variables are depicted on the x-axis by Y = Year, T = Stream temperature during peak migration, P = Pacific decadal oscillation, S = Sea surface temperature, F = Peak stream flow, Tlag = Average stream temperature during the growth and development period, D = conspecific density (See SI text). The y-axis is the sum of the AICC weights from each model that included a given covariate and had a ΔAICC<10, and represents the relative support for each variable. Variables with relative importance = 1 are present in all best-supported models. For each species and life history, all candidate variables are included on the figure, even those with relatively less support. See Figure 1 caption for description of figure colors (Blue = Pink salmon even- and odd-years combined). Shaded panels represent migration timing from freshwater to saltwater. (TIFF) [file pone.0053807.s003.tiff]

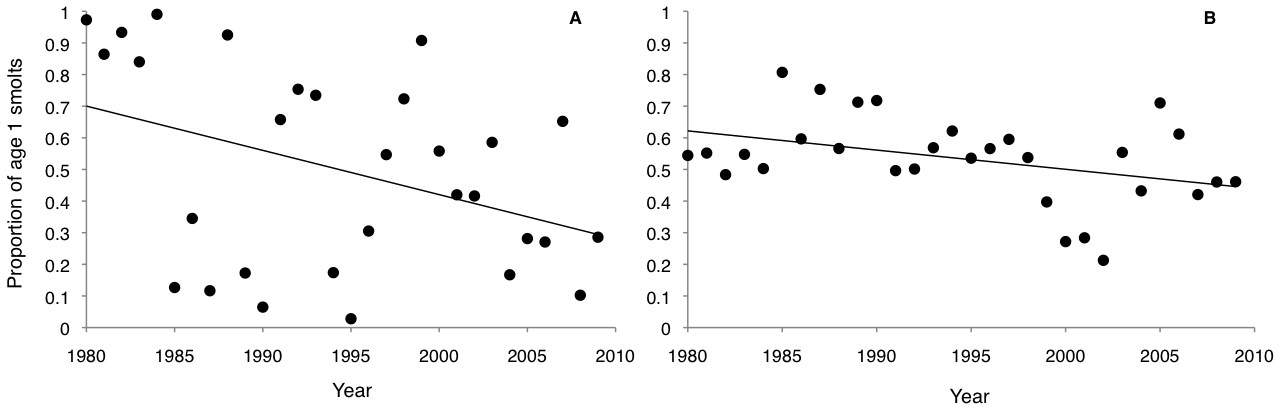

Supplement: Figure S4 — Proportion of age 1 smolts vs. time for sockeye (A) and coho (B). The fitted lines are linear regressions of the proportion of smolts vs. time (A, b1 = −0.014, SE = 0.006) (B, b1 = −0.006, SE = 0. 0.002) salmon. (TIFF) [file pone.0053807.s004.tiff]
