# Supplementary material for: Earlier Migration Timing, Decreasing Phenotypic Variation, and Biocomplexity in Multiple Salmonid Species
Source: PLoS One. 2013 Jan 10;8(1):e53807. doi: 10.1371/journal.pone.0053807 (PMC3542326; doi:10.1371/journal.pone.0053807)
Supplement: Table S1 — Results from the linear regressions of timing of migration and intra-annual trait variation vs. year. Regression describes which data were used in the analysis, n = sample size, b = slope from the regression, SE(b) = standard error of the slope, L CI = lower 95% confidence interval, U CI = upper 95% confidence interval, r 2 = coefficient of determination, P = P value of the regression analysis. (DOCX) [file pone.0053807.s005.docx]

| Table S1. Results from the linear regressions of timing of migration and intra-annual trait variation vs. year. Regression describes which data were used in the analysis, *n* = sample size, *b* = slope from the regression, SE(*b*) = standard error of the slope, L CI = lower 95% confidence interval, U CI = upper 95% confidence interval, *r*^2^ = coefficient of determination, *P* = *P* value of the regression analysis. | | | | | | |
| --- | --- | --- | --- | --- | --- | --- |
| Species/life history | Regression | *n* | *b* | SE(*b*) | R^2^ | *P* |
| Pink salmon adults | Median | 20 | -0.253 | 0.12 | 0.19 | 0.057 |
| odd year | Range | 20 | -0.340 | 0.12 | 0.30 | 0.013 |
|  |  |  |  |  |  |  |
| Pink salmon adults | Median | 20 | -0.331 | 0.11 | 0.32 | 0.010 |
| even year | Range | 20 | -0.227 | 0.12 | 0.17 | 0.068 |
|  |  |  |  |  |  |  |
| Sockeye adults | Median | 47 | 0.192 | 0.13 | 0.05 | 0.147 |
|  | Range | 47 | -0.311 | 0.21 | 0.05 | 0.150 |
|  |  |  |  |  |  |  |
| Sockeye jacks | Median | 40 | -0.305 | 0.16 | 0.09 | 0.059 |
|  | Range | 36 | -0.189 | 0.24 | 0.02 | 0.432 |
|  |  |  |  |  |  |  |
| Coho adults | Median | 39 | -0.418 | 0.07 | 0.51 | 0.000 |
|  | Range | 39 | -0.002 | 0.13 | 0.00 | 0.990 |
|  |  |  |  |  |  |  |
| Coho jacks | Median | 40 | -0.307 | 0.06 | 0.39 | 0.000 |
|  | Range | 39 | 0.366 | 0.09 | 0.29 | 0.000 |
|  |  |  |  |  |  |  |
| Pink salmon fry | Median | 19 | -0.273 | 0.17 | 0.13 | 0.132 |
| even year | Range | 19 | -0.278 | 0.12 | 0.24 | 0.034 |
|  |  |  |  |  |  |  |
| Pink salmon fry | Median | 19 | -0.494 | 0.15 | 0.39 | 0.004 |
| odd year | Range | 19 | -0.214 | 0.14 | -0.02 | 0.139 |
|  |  |  |  |  |  |  |
| Coho smolts age 1 | Median | 30 | 0.070 | 0.09 | 0.02 | 0.419 |
|  | Range | 30 | 0.214 | 0.08 | 0.19 | 0.016 |
|  |  |  |  |  |  |  |
| Coho smolts age 2 | Median | 30 | -0.091 | 0.09 | 0.04 | 0.314 |
|  | Range | 30 | -0.192 | 0.13 | 0.07 | 0.147 |
|  |  |  |  |  |  |  |
| Sockeye smolts age 1 | Median | 30 | 0.105 | 0.15 | 0.02 | 0.498 |
|  | Range | 30 | -0.064 | 0.13 | 0.01 | 0.631 |
|  |  |  |  |  |  |  |
| Sockeye smolts age 2 | Median | 30 | -0.140 | 0.16 | 0.03 | 0.377 |
|  | Range | 30 | -0.312 | 0.09 | 0.29 | 0.002 |
|  |  |  |  |  |  |  |
| Dolly Varden | Median | 31 | -0.070 | 0.12 | 0.01 | 0.558 |
|  | Range | 31 | -0.327 | 0.11 | 0.24 | 0.005 |
|  |  |  |  |  |  |  |
| Cutthroat trout | Median | 31 | -0.119 | 0.12 | 0.03 | 0.338 |
|  | Range | 31 | 0.392 | 0.24 | 0.08 | 0.115 |
