# Supplementary material for: Earlier Migration Timing, Decreasing Phenotypic Variation, and Biocomplexity in Multiple Salmonid Species
Source: PLoS One. 2013 Jan 10;8(1):e53807. doi: 10.1371/journal.pone.0053807 (PMC3542326; doi:10.1371/journal.pone.0053807)
Supplement: Table S2 — Model selection results for migration timing from saltwater to freshwater. AICC values for the models predicting the median date of migration timing from saltwater to freshwater for reproductively mature Pacific salmon. The model with the lowest AICC is highlighted in yellow. Y = year, T = temperature during migration, P = PDO, S = sea surface temperature, F = peak stream flow. (DOCX) [file pone.0053807.s006.docx]

| Table S2. Model selection results for migration timing from saltwater to freshwater. AIC_C_ values for the models predicting the median date of migration timing from saltwater to freshwater for reproductively mature Pacific salmon. The model with the lowest AIC_C_ is highlighted in yellow. *Y* = year, *T* = temperature during migration, *P* = PDO, *S* = sea surface temperature, *F* = peak stream flow. | | | | | | |
| --- | --- | --- | --- | --- | --- | --- |
| Model | Pink salmon | Coho adults | Coho jacks | Sockeye adults | | Sockeye jacks |
| *Null* | 271.66 | 261.62 | 256.69 | 372.42 | 314.18 | |
| *Y* | 262.43 | 235.79 | 239.19 | 372.30 | 312.47 | |
| *T* | 268.00 | 263.37 | 258.79 | 369.51 | 313.55 | |
| *PDO* | 272.96 | 263.57 | 258.64 | 367.68 | 316.18 | |
| *SST* | 273.62 | 259.74 | 254.90 | 373.54 | 316.29 | |
| *PF* | 267.30 | 244.94 | 254.47 | 372.40 | 315.88 | |
| *Y+T* | 252.42 | 230.65 | 239.16 | 371.17 | 313.31 | |
| *Y+P* | 264.42 | 238.01 | 241.38 | 369.26 | 314.27 | |
| *Y+S* | 264.34 | 233.31 | 237.04 | 373.69 | 314.69 | |
| *Y+F* | 261.38 | 230.82 | 241.41 | 372.43 | 314.38 | |
| *T+P* | 267.36 | 265.24 | 260.87 | 365.48 | 315.62 | |
| *T+S* | 269.69 | 261.96 | 256.53 | 369.75 | 315.75 | |
| *T+F* | 266.37 | 247.07 | 256.60 | 370.26 | 315.49 | |
| *P+S* | 274.68 | 261.56 | 256.76 | 369.50 | 318.40 | |
| *P+F* | 268.90 | 247.07 | 256.58 | 366.31 | 318.05 | |
| *T+F* | 269.52 | 246.90 | 255.03 | 372.22 | 318.05 | |
| *Y * T* | 254.45 | 229.86 | 237.04 | 372.69 | 313.06 | |
| *Y*F* | 262.98 | 230.06 | 243.74 | 374.30 | 311.77 | |
| *Y+T+P* | 252.38 | 232.47 | 241.15 | 367.83 | 315.25 | |
| *Y+T+S* | 253.57 | 230.91 | 238.63 | 371.77 | 315.64 | |
| *Y+T+PF* | 254.38 | 228.24 | 241.35 | 371.98 | 315.42 | |
| *Y+P+S* | 266.20 | 235.63 | 239.20 | 371.23 | 316.58 | |
| *Y+P+F* | 263.51 | 233.17 | 243.73 | 368.16 | 316.41 | |
| *Y+S+F* | 263.66 | 231.56 | 238.53 | 372.63 | 316.65 | |
| *T+P+S* | 269.57 | 263.88 | 258.69 | 366.74 | 317.93 | |
| *T+P+F* | 266.64 | 249.24 | 258.88 | 365.10 | 317.74 | |
| *T+S+F* | 268.01 | 249.22 | 256.85 | 369.09 | 317.74 | |
| *Y+T+P+S* | 254.27 | 232.78 | 240.71 | 369.23 | 317.66 | |
| *Y+T+P+F* | 254.68 | 230.33 | 243.44 | 367.58 | 317.58 | |
| *Y+T+S+F* | 255.63 | 230.14 | 239.90 | 371.25 | 317.79 | |
| *Y+P+S+F* | 265.82 | 234.04 | 240.78 | 369.05 | 318.77 | |
| *T+P+S+F* | 268.85 | 251.51 | 259.22 | 365.02 | 320.11 | |
| *Y+T+P+S+F* | 256.68 | 232.32 | 241.95 | 367.65 | 320.05 | |
