# Supplementary material for: Earlier Migration Timing, Decreasing Phenotypic Variation, and Biocomplexity in Multiple Salmonid Species
Source: PLoS One. 2013 Jan 10;8(1):e53807. doi: 10.1371/journal.pone.0053807 (PMC3542326; doi:10.1371/journal.pone.0053807)
Supplement: Table S3 — Model selection results for migration from freshwater to saltwater. AICC values for the models predicting the median date of migration timing from freshwater to saltwater for the various species and life histories. The model with the lowest AICC is highlighted in yellow. Y = year, T = temperature during migration, Tlag = temperature during the developmental period leading up to migration, D = conspecific density (See covariate descriptions). (DOCX) [file pone.0053807.s007.docx]

| Table S3. Model selection results for migration from freshwater to saltwater. AIC_C_ values for the models predicting the median date of migration timing from freshwater to saltwater for the various species and life histories. The model with the lowest AIC_C_ is highlighted in yellow. *Y* = year, *T* = temperature during migration, *Tlag* = temperature during the developmental period leading up to migration, *D* = conspecific density (See covariate descriptions). | | | | | | | |
| --- | --- | --- | --- | --- | --- | --- | --- |
| Model | Pink salmon | Coho age 1 | Coho age 2 | Sockeye age 1 | Sockeye age 2 | Dolly Varden | Cutthroat trout |
| Null | 275.96 | 171.93 | 174.42 | 206.28 | 207.83 | 200.35 | 202.65 |
| Y | 267.50 | 173.36 | 175.46 | 207.93 | 209.12 | 202.11 | 203.79 |
| T | 240.02 | 158.58 | 138.88 | 205.00 | 200.44 | 172.46 | 172.34 |
| Tlag | 262.53 | 170.10 | 166.04 | 207.71 | 209.91 | 194.23 | 196.23 |
| D |  | 171.07 | 176.55 | 208.08 | 209.82 | 200.37 | 203.75 |
| Y+T | 225.68 | 158.99 | 139.04 | 206.55 | 201.96 | 174.74 | 174.11 |
| Y+Tlag | 258.57 | 170.33 | 168.16 | 209.73 | 211.42 | 196.52 | 198.39 |
| Y+D |  | 173.09 | 176.23 | 210.03 | 211.03 | 202.34 | 205.58 |
| T+Tlag | 238.59 | 160.54 | 136.94 | 203.39 | 200.96 | 172.88 | 172.62 |
| T+D |  | 156.97 | 141.08 | 206.19 | 201.27 | 173.68 | 173.00 |
| Tlag + D |  | 169.01 | 168.33 | 209.93 | 211.94 | 195.01 | 198.25 |
| Y * T | 228.04 | 161.44 | 141.33 | 202.93 | 203.19 | 177.16 | 176.30 |
| Y * Tlag | 256.36 | 172.68 | 170.62 | 201.31 | 209.49 | 198.73 | 200.82 |
| T*Tlag | 239.98 | 162.99 | 138.79 | 205.75 | 201.07 | 175.28 | 175.08 |
| Y+T+Tlag | 226.97 | 160.54 | 138.22 | 205.66 | 201.83 | 175.30 | 174.89 |
| Y+T+D |  | 159.45 | 140.39 | 208.23 | 202.29 | 176.13 | 175.38 |
| Y+Tlag+D |  | 171.46 | 170.26 | 212.16 | 213.44 | 197.46 | 200.67 |
| T+Tlag+D |  | 158.99 | 139.33 | 205.63 | 202.72 | 174.39 | 174.00 |
| Y+T+Tlag+D |  | 161.63 | 140.35 | 208.15 | 203.31 | 176.99 | 176.64 |
