# Supplementary material for: Earlier Migration Timing, Decreasing Phenotypic Variation, and Biocomplexity in Multiple Salmonid Species
Source: PLoS One. 2013 Jan 10;8(1):e53807. doi: 10.1371/journal.pone.0053807 (PMC3542326; doi:10.1371/journal.pone.0053807)
Supplement: Table S4 — Correlations between environmental variables and time (Year) for those data used to predict migration timing for each species and life history. All values are Pearson product moment correlations. Values above the double horizontal line are for data used to predict median date of migration timing into freshwater from the ocean, and values below are for data used to predict salmonid migration timing from freshwater into saltwater. T refers to water temperatures during peak migration timing, F refers to flows during peak migration timing, PDO refers to values of the Pacific Decadal Oscillation, SST refers to sea-surface temperature, and Tlag refers to temperatures during developmental periods in freshwater (see Text S1 for more information). The label “All” refers to data used for all species and life histories migrating into saltwater except for pink salmon. (DOCX) [file pone.0053807.s008.docx]

| Table S4. Correlations between environmental variables and time (Year) for those data used to predict migration timing for each species and life history. All values are Pearson product moment correlations. Values above the double horizontal line are for data used to predict median date of migration timing into freshwater from the ocean, and values below are for data used to predict salmonid migration timing from freshwater into saltwater. T refers to water temperatures during peak migration timing, F refers to flows during peak migration timing, PDO refers to values of the Pacific Decadal Oscillation, SST refers to sea-surface temperature, and Tlag refers to temperatures during developmental periods in freshwater (see Text S1 for more information). The label “All” refers to data used for all species and life histories migrating into saltwater except for pink salmon. | | | |
| --- | --- | --- | --- |
|  | Year | Flow | PDO |
| Pink T | 0.1492029 | 0.3269946 | 0.2577341 |
| Coho T | 0.3070841 | 0.0883677 | 0.2485382 |
| Sockeye T | 0.3703783 | 0.1443037 | 0.1126701 |
|  |  |  |  |
| Pink F | 0.02640422 |  | -0.07258238 |
| Coho F | -0.3127213 |  | -0.03700868 |
| Sockeye F | -0.5028006 |  | -0.1098793 |
|  |  |  |  |
| Pink PDO | 0.1532378 | -0.07258238 |  |
| Coho PDO | 0.05972725 | -0.03700868 |  |
| Sockeye PDO | 0.3022053 | -0.1098793 |  |
|  |  |  |  |
| Pink SST | 0.03254216 | 0.1684941 | 0.3125356 |
| Coho SST | -0.08595782 | 0.4070197 | 0.09457787 |
| Sockeye SST | -0.0712369 | 0.3015931 | -0.1616757 |
|  |  |  |  |
|  | Year | Temp |  |
| Pink T | 0.1734565 |  |  |
| All T | 0.3263582 |  |  |
|  |  |  |  |
| Pink Tlag | 0.3374658 | 0.5330291 |  |
| All Tlag | 0.480725 | 0.5431625 |  |
