# Supplementary material for: Earlier Migration Timing, Decreasing Phenotypic Variation, and Biocomplexity in Multiple Salmonid Species
Source: PLoS One. 2013 Jan 10;8(1):e53807. doi: 10.1371/journal.pone.0053807 (PMC3542326; doi:10.1371/journal.pone.0053807)
Supplement: Text S1 — The supplementary text provides a description of each species life history, and a detailed description of the environmental variables used in model selection analyses. (DOCX) [file pone.0053807.s009.docx]

**Text S1**

*Auke Creek salmonids: life history description and data preparation*

Pink salmon

Pink salmon (*Oncorhynchus gorbuscha*) have the simplest life history of the salmonids in Auke Creek. Pink salmon have a strict semelparous two-year life cycle. The result of this life history is that odd- and even-year pink salmon have undergone different demographic and evolutionary histories and are reproductively isolated (Churikov & Gharrett 2002), and we treated them as independent populations. Odd- and even- year populations refer to the year in which adults spawn. Pink salmon enter Auke Creek to spawn in late July through the middle of September. The following spring, juveniles migrate to the ocean. Pink salmon spend approximately one and a half years in the ocean before returning to Auke Creek to spawn. Although pink salmon have a simple life cycle, they demonstrate important biocomplexity and local adaptation in the form of migration timing diversity (Smoker et al. 1998, Gharrett et al. 2001).

Alexansdottir and Mathisen (1982) documented selective harvest impacts on Southeast Alaska pink salmon run timing and population productivity on a broad regional scale spanning the time period from 1926-1944. Specifically, fish traps focused tremendous harvest pressure on early returning mixed stock fisheries in the major migratory paths of pink salmon in Southeast Alaska. Since 1945 there have been regulations to ensure that harvest occurs across the migratory timing of pink salmon in an effort to reduce potential selective effects.

Coho Salmon

Coho salmon (*Oncorhynchus kisutch*) are semelparous and migrate into Auke Creek to spawn from September through October. Juveniles spend either one or two years in Auke Lake before migrating to the ocean as smolts in May and June. The amount of time spent in freshwater is a function of both genetic and environmental influences (Quinn 2005). The largest smolts (age 2) tend to migrate earliest while smaller smolts (age 1) migrate later in the season. Scale samples for aging are collected from approximately 50 individuals (each day) from 4-12 days of the outmigration. For each year, we used logistic regression and scale aging data to estimate the proportion of age 1 and age 2 smolts that emigrated each day. The term for an effect of time in the logistic model was significant (*P* < 0.05) in 27 out of 30 years, suggesting that this model was an adequate representation of the differences in migration timing between age 1 and age 2 smolts. In years in which time did not influence age proportions (1982, 1983, 1985), daily proportions of age 1 and 2 smolts were estimated from the overall mean of the scale aging data. Excluding these data points had negligible effects on the results.

As an alternative life history, some male coho salmon spend one summer (approximately one half year) in the ocean before returning to Auke Creek to spawn. Individuals with this life history are referred to as “jacks”. Other coho salmon spend one and a half years in the ocean before returning to Auke Creek. In Alaska, Auke Creek is the only location that provides complete counts of jacks. Therefore, Auke Creek is critical to investigations that attempt to understand how biocomplexity influences demographic and evolutionary trajectories for this species. Importantly, alternative life histories in Pacific salmon are often a function of both genetic and environmental variation (Quinn 2005, Hutchings 2011).

Sockeye salmon

Auke Creek sockeye salmon (*Oncorhynchus nerka*) juvenile life history and phenology are similar to those of coho salmon (see above). Scale samples are also collected from sockeye salmon smolts, and we used these data to estimate proportions of age 1 and age 2 smolts, as described above. The sample size (number of days that scales were collected and aged) was insufficient (< 4 days) to fit a logistic model in five years. In the remaining years, time significantly (*P* < 0.05) influenced intra-annual age proportions in 21 of 25 years. In years where the time was not a significant effect, it was largely due to a strong cohort effect, where either age 1 or age 2 smolts were much more abundant than the other age class. As described above, we used mean age proportions when samples sizes were limited or when a logistic model did not fit the data.

Adult sockeye salmon are semelparous and enter Auke Creek in July and August. Similar to coho, some male sockeye adopt the “jack” alternative life history. Jacks spend slightly more than one year in the ocean. Other sockeye spend between 2-5 years in the ocean before they return to Auke Creek as reproductively mature adults. To estimate the trend in phenotypic variation we did not use data from 1963, 1964, and 1968-1970 because sampling did not include the latest migrating fish and therefore negatively biased the data in these years. Generally, the vast majority of fish migrate during 1-2 days; but this bias does not influence the value for the median date of migration timing as long as the peak dates are sampled, which they were.

DollyVarden

Dolly Varden char (*Salvelinus malma*) are fall spawning, iteroperous fish, which migrate into Auke Creek throughout the fall. It is unknown if Dolly Varden successfully spawn in the system, though adult fish are present and appear to be reproductively mature based on phenotypic characteristics. Reproductively immature fish also migrate into Auke Creek during the fall and use Auke Lake as a wintertime refugium. Most fish migrate back to the ocean the following spring, usually during April and May. The number of times they migrate back and forth from freshwater to saltwater and the duration of time they spend in either habitat appears to be variable and is generally unknown. Only data on migration of Dolly Varden to saltwater are available.

There is a strong, positive relationship between timing of emigration and individual size, in which the largest individuals migrate earliest. Large cohort effects could potentially obscure different trends in migration timing for different age classes if they are not accounted for. A proportion of fish was measured (fork length to the nearest 5 mm) on most days during the course of the Dolly Varden emigration. We used these data to estimate overall age proportions with logistic models based on size categories. Logistic models significantly (*P* < 0.05) fit the data in 28 of 30 years. Based on visual inspections of the length frequency distributions in multiple years and our basic understanding of the biology of Dolly Varden in Auke Creek, we used three size categories; 1-180mm, 181-320mm, and >320mm. For each year, we estimated the proportion of the smallest and the largest size classes with two separate logistic regressions, where logistic models estimated the proportion of small (or large) fish on each date relative to the proportion of all other fish. We then estimated the proportion of individuals in the middle size class by subtracting the estimated proportions of the largest and smallest size classes from 1.0. We conducted linear regressions of the trend in phenology and trait variation vs. time from both the estimated age proportion data and the pooled (all size classes) data. There were minimal quantitative or qualitative differences between the results, so we presented only the pooled data in the primary findings. Dolly Varden are used for subsistence purposes and are economically valuable because of recreational fisheries, but they are not targeted in commercial fisheries.

Coastal cutthroat trout

Coastal cutthroat trout (*Oncorhynchus clarki clarki*) are iteroperous and have a very complex life history. Similar to Dolly Varden, cutthroat trout primarily use Auke Creek and Auke Lake as a wintertime refuge. It is unknown if they reproduce in the system. Cutthroat trout may spend varying amounts of time in the system, from one winter to multiple years. Most cutthroat trout migrate into Auke Creek during the fall and leave the system during the spring. Similar to Dolly Varden, the largest individuals migrate earliest in each year. These individuals are often reproductively mature and migrate to various other stream systems near Auke Creek to spawn (Jones and Seifert 1997). Because large individuals are generally reproductively active (as opposed to migrating to the ocean to feed on marine resources), we assumed that these ecological differences might be reflected in terms of different temporal trends in migration timing.

Data on cutthroat trout migration timing to saltwater were available beginning in 1980. Length was measured for almost every individual emigrating from Auke Creek, except in 1980, 1981, 1983, 1985, and 2001. Based on visual inspections of length frequency distributions and our basic knowledge of cutthroat trout biology in Auke Creek, we used these data to separate the cutthroat trout data into three size classes (small <191mm, medium 191-320mm, large >320mm). Because nearly every individual fish is measured, we did not use logistic regressions to estimate size (age) proportions. Similar to Dolly Varden, using age structure data as opposed to the entire pooled size distribution did not change the results of the analysis. The pooled cutthroat trout emigration data are presented in the primary findings. Cutthroat trout are used for subsistence purposes and are economically valuable because of recreational fisheries, but they are not targeted in commercial fisheries.

**Identifying variables related to migration timing**

Environmental covariates used for model selection analyses:

*Water temperature*

Low stream flows and high stream temperatures appear to be negatively correlated with migration timing in adult salmon that migrate after peak annual summer water temperatures (Robards & Quinn 2002, Quinn 2005, Goniea et al. 2006). Daily stream temperatures have been measured at Auke Creek since 1963. Daily stream flow data have only been measured intermittently at Auke Creek; however, intra-annual stream temperature variation is strongly correlated with stream discharge (*r* = -0.646, Fukushima and Smoker 1997). Therefore, stream temperature was used as an index of local abiotic conditions at the site of spawning/migration for adult salmon. Average stream temperatures during the peak period of migration timing were used to describe local conditions encountered by the majority of adult migrating pink salmon (August 20 – September 10), coho salmon (September 15 – October 10), and sockeye salmon (July 1 – July 31).

We hypothesized that temperature could influence migration timing from freshwater to saltwater by two mechanisms. First, temperature may influence migration timing by influencing cumulative growth and development in freshwater such that warm temperatures over an extended period of time increase growth and development causing individual fish to migrate earlier (Groot & Margolis 1991, Quinn 2005). Second, many of the important physiological and developmental changes associated with migration from freshwater to saltwater occur immediately prior to the migratory event (Groot & Margolis 1991, Hodgson et al. 2006). Therefore, temperatures during the time period immediately prior to migration may be important in determining inter-annual variation in timing. Also, migration timing from saltwater to freshwater is associated with the temporal availability of food resources (pink, sockeye, coho salmon, Dolly Varden char, immature cutthroat trout) or spawning (mature cutthroat trout). Therefore, temperatures immediately prior to and during the migration event may act as a cue for phenological events in other species (i.e. prey) or environmental conditions in other locations. To approximate the first mechanism we used average stream temperatures during the previous year. Specifically, average stream temperature from June – March was used for Dolly Varden, cutthroat trout, sockeye, and coho salmon and temperatures from September – February were used for pink salmon. For the second mechanism, we used average stream temperatures during the period of peak outmigration timing for each species (April for pink salmon and May for all other species). Though there was marginal correlation (*r* = 0.5) between these variables, we included both in linear models because they are not redundant in a statistical or biological sense (Burnham & Anderson 2002).

*Stream Flow*

Based on our own observations, we hypothesized that sockeye salmon (adults and jacks) and pink salmon migrate into Auke Creek during the first major flow event during their peak reproductive period. To obtain values for this covariate we used precipitation records because precipitation is highly correlated with stream flow (one-day lag *r* = 0.865). NOAA has maintained a nearly complete record of daily precipitation near the mouth of Auke Creek since 1963. During the peak migratory time period for sockeye and pink salmon, we used the first date that 2 inches of rain were measured during a four-day period or the middle date from the 7-day period with greatest precipitation, whichever came first. Stream flows during the coho migration are generally higher, and we used the middle date from the 7-day period with greatest precipitation as a covariate for this species (adults and jacks). Our variable for peak flow was only marginally correlated with stream temperature (Pearson’s correlation *r* = 0.327 pink, *r* = 0.088 coho, *r* = 0.144 sockeye salmon).

*Oceanic conditions*

Oceanic conditions during the spring and summer before spawning can influence migration timing (Hodgson et al. 2006, Crozier et al. 2011, Mundy & Evensen 2011), potentially by influencing reproductive development (Groot & Margolis 1991), or acting as an indirect cue/indicator for migration conditions in estuaries of freshwater (e.g. Dahl et al 2004, Hodgson 2006). It is unclear if temperature has a gradual effect over longer periods of time (and by necessity larger geographic space) or if conditions immediately prior to migration and during the final and most dramatic physiological changes are of most importance. Sea-surface temperatures were obtained from the International Comprehensive Ocean-Atmosphere Date Set. Values were obtained from two 1° x 1° grid boxes located at 58°S-59°N and –(134°E-136°W). Overall mean values for the month that each salmon species migrates into Auke Creek were computed from data within these grids (pink salmon = August, coho = September, sockeye = June and July). This location includes the area immediately around Auke Bay and the ocean area used by each salmon species in their final migration towards Auke Creek.

The Pacific Decadal Oscillation (PDO) is a composite measure of oceanic temperature conditions and is correlated with sea-surface temperatures and productivity in the North Pacific (Mantua et al. 1997). Therefore, the PDO provides an index of broad scale oceanic conditions that may influence migration timing. PDO measurements were obtained from the University of Washington (<http://jisao.washington.edu/pdo/PDO.latest>). The mean of the monthly PDO values from March-June were used for sockeye salmon, April-July were used for pink salmon and May-August for coho salmon. This time period is important for final somatic and gametic development and is related to migration timing in sockeye salmon across the Northern Pacific (Hodgson et al. 2006).

*Density*

Density dependent growth and survival for salmonids in freshwater is well documented (Quinn 2005, Groot & Margolis 1991). We hypothesized that increased density could delay migration timing from freshwater to saltwater if growth and development are limited by density interactions (Reed et al. 2010). We used the overall number of migrating fish of each species as our measurement of density.

**Supplementary References**

Alexandersdottir M, Mathisen O (1982) Changes in Southeast Alaska Pink Salmon Populations, 1914-1960. Fisheries Research Institute, University of Washington,

Burnham KP, Anderson DR (2002) Model selection and multimodel inference: a practical information theoretic approach Springer, New York.

Churikov D, Gharrett AJ (2002) Comparative phylogeography of the two pink salmon broodlines: an analysis based on mitochondrial DNA genealogy. Mol Ecol 11: 1077-1011.

Crozier LG, Scheuerell MD, Zabel RW (2011) Using time series to characterize evolutionary and plastic responses to environmental change: a case study of a shift toward earlier migration date in sockeye salmon. Am Nat 78: 755-773.

Dahl J, Dannewitz J, Karlsson L, Petersson E, Löf A, & Ragnarsson B (2004) The timing of spawning migration: implications of environmental variation, life history, and sex. Can J Zool 82: 1864-1870.

Fukushima M, Smoker WW (1997) Determinants of stream life, spawning efficiency, and spawning habitat in pink salmon in the Auke Lake system, Alaska. Can J Fish Aquat Sci 54: 96-104.

Gharrett AJ, Lane S, McGregor AJ, Taylor SG (2001) Use of a genetic marker to examine genetic interaction among subpopulations of pink salmon (*Oncorhynchus gorbuscha*). Genetica 111: 259-267.

Goniea TM, Keefer ML, Bjornn TC, Peery CA, Bennett DH (2006) Behavioral thermoregulation and slowed migration by adult fall Chinook salmon in response to high Columbia River water temperatures. Tran Am Fish Soc 135: 408-419.

Groot C, Margolis L (1991) *Pacific salmon life histories*. University of British Columbia Press, British Columbia.

Hodgson S, Quinn TP, Hilborn R, Francis RC, Rogers DE (2006) Marine and freshwater climatic factors affecting interannual variation in the timing of return migration to freshwater of sockeye salmon (*Oncorhynchus nerka*). Fish Ocean 15: 1-24.

Hutchings JA (2011) Old wine in new bottles: reactions norms in salmonid fishes. Heredity 106: 421-437.

Jones JD, Seifert CL (1997) Distribution of mature sea-run cutthroat trout overwintering in Auke Lake and Lake Eva in Southeastern Alaska. In ‘Sea-run cutthroat trout: biology, management, and future conservation’ pp. 27-28. Oregon Chapter, Am Fish Soc.

Mantua NJ, Hare SR, Zhang Y, Wallace JM, Francis RC (1997) A Pacific interdecadal climate oscillation with impacts on salmon production. Bull Amer Meteor Soc 78: 1069-1079.

Mundy PR, Evenson DF (2011) Environmental controls of phenology of high-latitude Chinook salmon populations of the Yukon River, North America, with application to fishery management. ICES J Mar Sci 68: 1155-1164.Quinn, T.P. (2005). *The Behavior and Ecology of Pacific Salmon and Trout*. University of Washington Press, Seattle.

Reed TE, Martinek G, Quinn TP (2010) Lake-specific variation in growth, migration timing and survival of juvenile sockeye salmon *Oncorhynchus nerka*: separating environmental from genetic influences. J Fish Bio 77: 692-705.

Robards MD, Quinn TP (2002) The migratory timing of adult summer-run steelhead trout (Oncorhynchus mykiss) in the Columbia River: Six decades of environmental change. Tran Am Fish Soc 131: 523-536.

Smoker WW, Gharrett AJ, Stekoll MS (1998) Genetic variation of return date in a population of pink salmon: a consequence of fluctuating environment or dispersive selection? Alaska Fish Res Bull 5:46-54.
